# Healthy aging in the Americas

**DOI:** 10.26633/RPSP.2021.116

**Published:** 2021-09-01

**Authors:** Jarbas Barbosa da Silva, John W. Rowe, Jose Ricardo Jauregui

**Affiliations:** 1 Pan American Health Organization Washington, D.C. United States of America Pan American Health Organization, Washington, D.C., United States of America; 2 International Association of Gerontology and Geriatrics, and Mailman School of Public Health, Columbia University New York United States of America International Association of Gerontology and Geriatrics, and Mailman School of Public Health, Columbia University, New York, United States of America New York, United States of America; 3 International Association of Gerontology and Geriatrics New York United States of America International Association of Gerontology and Geriatrics, New York, United States of America

**Keywords:** Aging, healthy aging, Latin America, Caribbean region, Americas

In just the past 50 years, life expectancy has increased by more than 20 years. This significant increase in longevity is due in part to advances in medicine, public health interventions, biotechnology, and social and economic development that have made it possible for people to live longer than at any other time in history (1).

In Latin America and the Caribbean (LAC), the proportion of the population aged 60 and over will increase by up to 18% over the next decade, reaching between 25% and 30% by 2050. This transition will occur in 35 years, just half the time it took in the United States and Canada (2). With this rapid demographic transition, the so-called “window of demographic opportunity”, which is the time to prepare for the demographic transition, is rapidly narrowing in the Region of the Americas. Although the sense of urgency in prioritizing aging has grown, greater efforts should be made to address this impending demographic shift. Action and targeted interventions are required to ensure that longevity and aging are positive outcomes of sustainable development in the Americas (3). Demographic shifts, along with epidemiological transitions and other challenges such as migration and climate change, require countries to create innovative ways across all sectors to address these new realities (4).

Despite the predictability of population aging, the world is far from prepared to address this demographic transition. The COVID-19 pandemic has shed a light on many existing gaps in what we do and in the way we think about aging and older people. The pandemic has exposed how widespread ageism—stereotypes, prejudices and discrimination based on age—is in society, particularly in health systems and health service organizations. Many decisions associated with care, use of health systems resources, and measurements to contain the virus’ spread, such as discussions around vertical isolation, were based solely on chronological age (5).

The current situations observed during the COVID-19 pandemic along with the longevity revolution in the Region is an invitation to build a new vision for the future, to visualize a world in which older persons are valuable, active and productive members of the society (6). In this context and with the aim to improve the life of older persons, the Decade of Healthy Aging (2021-2030) was approved by the World Health Assembly (WHA) in August 2020 and proclaimed by the United Nations General Assembly (UNGA) in December 2020. The Decade of Healthy Aging is the main strategy to achieve and support actions to build a society for all ages with the vision of promoting a world in which everyone can live a long and healthy life. This strategy requires collective and concerted action over the next decade by governments, international and regional organizations, civil society, the private sector, academia, and the media to ensure that older age is indeed an opportunity for all (7).

The Decade will focus on four main areas of action which include: Changing how we think, feel and act towards age and aging; ensuring communities foster the abilities of older persons; delivering person-centered integrated care and primary health services responsive to older people; and providing access to long-term care for older people who need it (8).

It is further built upon four enablers that will contribute to the achievement of results in the four main action areas. One of these enablers is strengthening data, research, and innovation. Three quarters of the countries worldwide have limited or no data on healthy aging or on older age groups. In 2020, in the Region of the Americas, only 15 countries reported having cross-sectional, nationally representative data on the health status and needs of older persons, and only 10 reported having longitudinal representative studies of this nature in the public domain. This lack of research contributes to the invisibility and exclusion of older people. To fully address this heterogenous group, data must be disaggregated to better understand older persons’ health status, social and economic wellbeing, and community connections (1). There has been a lack of gathering and reporting disaggregated data by age, even in situations that older adults have higher vulnerability, such as emergencies, disasters, and different chronic conditions. For example, during the COVID-19 pandemic, there was a lack of disaggregated data by age for reported cases or deaths, especially in low- and middle-income countries.

Research on healthy aging must address the existing gaps and current needs of older people, anticipate future challenges, and link the social, biological, economic and environmental conditions and determinants of healthy aging in the first and the second halves of life and develop interventions to improve healthy aging trajectories (8). Enhancing research knowledge of older adults and healthy aging and creating a solid scientific base on this matter will contribute to informed and evidence-based decision-making; and to evaluate and promote cost-effective interventions that generate important improvements in the health and well-being of aging societies (3).

To bring into focus the importance of research on healthy aging in the Region of the Americas and present relevant research from the Region, the Pan American Health Organization (PAHO) has developed this special edition of the *Pan American Journal of Public Health* on healthy aging, focusing on Latin America. In the context of the Decade of Healthy Aging and recognizing the importance of collaboration to strength efforts on this area, the Pan American Health Organization has worked with the International Association of Gerontology and Geriatrics (IAGG) in the development of this special edition, in line with its mission to promote the highest levels of achievement in gerontological research, and to interact with other organizations in the promotion of gerontological interests.

## References

[1] 1. World Health Organization. Decade of healthy ageing: Baseline report. Geneva: WHO; 2020 [accessed August 2 2021]. Available at: https://apps.who.int/iris/handle/10665/338677

[2] 2. Pan American Health Organization. Health in the Americas+, 2017 edition. Summary: Regional outlook and country profiles. Washington, DC: PAHO; 2017.

[3] 3. Vega Garcia E. Research: A priority for the decade of healthy aging. Colomb Med. 2019;50(2):50−1. [accessed August 2 2021] Available at: 10.25100/cm.v50i2.4613 PMC6774578 31607762

[4] 4. United Nations, Department of Economic and Social Affairs, Population Division. World population ageing 2019: Highlights. New York: UN; 2019 (ST/ESA/SER.A/430).

[5] 5. World Economic Forum. Time for the world to invest in healthy ageing. Davos: WEF; 2021 [accessed August 2 2021]. Available at: https://www.weforum.org/agenda/2021/01/invest-un-decade-healthy-ageing/

[6] 6. Guillén MF. 2030: How today’s biggest trends will collide and reshape the future of everything. New York: St. Martin’s Press; 2020.

[7] 7. World Health Organization. UN Decade of Healthy Ageing (2021-2030). Geneva; 2020 [accessed August 2 2021]. Available at: https://www.who.int/initiatives/decade-of-healthy-ageing

[8] 8. World Health Organization. Decade of Healthy Aging: Plan of action. Geneva; 2020 [accessed August 2 2021]. Available at: https://www.who.int/docs/default-source/decade-of-healthy-ageing/final-decade-proposal/decade-proposal-final-apr2020-en.pdf

